# Bacterial Isolates and Resistance Patterns in Preterm Infants with Sepsis in Selected Hospitals in Ethiopia: A Longitudinal Observational Study

**DOI:** 10.1177/2333794X20953318

**Published:** 2020-10-03

**Authors:** Beza Eshetu, Mulatu Gashaw, Semaria Solomon, Melkamu Berhane, Kassie Molla, Tamrat Abebe, Solomon Gizaw, Alemseged Abdissa, Mahlet Abayneh, Robert L. Goldenberg, Zemene Tigabu, Amha Mekasha, Bogale Worku, Elizabeth M. McClure, Assaye K. Nigusse, Lulu M. Muhe

**Affiliations:** 1 Jimma University College of Public Health and Medical Sciences, Jimma, Ethiopia; 2 St Paul Millennium Medical College Hospital, Addis Ababa, Ethiopia; 3 University of Gondar, Gondar, Ethiopia; 4 Addis Ababa University College of Health Sciences, Addis Ababa, Ethiopia; 5 Columbia University, NY, USA; 6 Ethiopian Pediatric Society, Addis Ababa, Ethiopia; 7 RTI International, Research Triangle Park, USA; 8 Bill & Melinda Gates Foundation, Seattle, WA, USA

**Keywords:** neonate, sepsis, multidrug resistance

## Abstract

*Background:* Neonatal sepsis is the third leading cause of neonatal mortality, behind prematurity and intrapartum-related complications. The main objectives of this study are to assess the proportion of sepsis in preterm newborns and identify the etiologic agents and their antibiotic sensitivity patterns. *Methods:* A longitudinal observational study was done from July 2016 to May 2018. Whenever clinical diagnosis of sepsis was made, blood cultures and antibiotic susceptibility tests were done. *Result:* We did 690 blood cultures, 255 (36.9%) showing bacterial growth. The most commonly isolated bacteria were *Klebsiella species* 78 (36.6%), *Coagulase negative Staphylococcus* 42 (19.7%) and *Staphylococcus aureus* 39 (18.3%). Gram-positive bacteria showed high resistance to penicillin (98.9%) and ceftriaxone (91.3%) whereas Gram-negative bacteria were highly resistant to gentamicin (83.2%) and ceftriaxone (83.2%). *Conclusion:* Resistance to the more commonly used antibiotics such as ampicillin and gentamycin was very high, necessitating reconsideration of the empiric use of these antibiotics.

## Background

Every year, 2.6 million neonates die globally; three fourths of these deaths occur in the first weeks of life, and almost all (99%) occur in low- and middle-income countries (LMICs).^1,2^ Neonatal sepsis is the third leading cause of neonatal mortality, only behind prematurity and intrapartum-related complications (or birth asphyxia).^3^ The World Health Organization (WHO) estimates that one million deaths per year are due to neonatal sepsis and that 42% of these deaths occur in the 1^st^ week of life.^2^ In addition, the survivors of neonatal sepsis are vulnerable to short and long-term neuro-developmental morbidity.^4-6^

Neonatal sepsis is a life-threatening condition and needs immediate empirical antimicrobial therapy to reduce mortality. It is important to choose an antibiotic regimen that covers the most common pathogens.^7^

Blood culture remains the gold standard for diagnosis of neonatal sepsis, despite its low sensitivity which may be due to small volume of the blood sample, or the use of empirical antibiotics prior to sampling.^8^ Neonatal sepsis is classically defined as the presence of symptoms of sepsis in the neonatal period combined with bacteriological isolation of an infectious agent from blood or cerebrospinal fluid (CSF).^9^ Early-onset neonatal sepsis refers to the presence of a confirmed infection in the blood or CSF of patients younger than 3 days of life, and late-onset neonatal sepsis refers to the onset of such infections between 3 and 28 days.^10^

The type of organisms causing sepsis varies from one region to another and changes over time even in the same place.^11,12^ This variation is often attributed to the changing pattern of antibiotic use and changes in lifestyle.^13,14^

In developing countries, clinically diagnosed sepsis occurs in 49-170 newborns per 1000 live births, whereas culture-proven sepsis occurs in 16 per 1000 live births.^15^The continuous evolution of drug resistance of pathogens causing neonatal sepsis is a major problem. Notably, methicillin resistant *S. aureus* (MRSA) and extended spectrum beta lactamase (ESBL) producing bacteria and multidrug resistant (MDR) Gram-negative organisms represent the principal setbacks to fighting infections. Most Gram-negative bacteria are now resistant to ampicillin and many are also becoming resistant to gentamicin.^16,17^

Although antimicrobial treatment is the cornerstone of the treatment of neonatal sepsis, due to the lack of local data on bacterial profiles and antimicrobial resistance patterns, there is a lack of clear consensus on the choice of antimicrobials for many settings in LMICs. Therefore, clinicians in LMICs are limited to use the data from the other settings, mostly from developed countries, to inform their treatment decisions.

## Objectives

The primary objective of this study was to assess the proportion of clinical and bacteriologically confirmed sepsis among preterm newborns admitted to selected hospitals in Ethiopia.

The secondary objectives of this study include:

- To determine common organisms causing sepsis in preterm newborns
- To determine antimicrobial susceptibility of the identified microorganisms

## Methodology

### Study Design

A multi-center longitudinal observational study design was used which included all preterm babies (<37 weeks of gestation) who were either delivered in or were referred to the study hospitals before 7 days of life from July 2016 to May 2018. Preterm newborns admitted at >7 days of life were excluded from the study as the original study used Ballard score (which is accurate before 7 days of life) to accurately estimate the gestational age of the babies for inclusion into the study. Thus, for this analysis, we included those neonates who were admitted to a NICU at <7 days. The preterm babies admitted to the NICUs were identified based on the study eligibility criteria by trained nurses. After screening, written informed consent was obtained from parents before enrolling the babies. The maternal medical history and physical examination including gestational age estimation was done by trained study physicians. Once a clinical diagnosis of sepsis was made (based on clinical signs including temperature instability, respiratory distress, decreased age appropriate neonatal reflexes, altered mentation, abnormal body movement or poor feeding), patients often underwent necessary laboratory investigations including complete blood count (CBC), blood culture, and CSF analysis and culture to establish the diagnosis of sepsis. Other investigations were also done as clinically indicated.^18^

### Study Settings

The study was conducted in five hospitals in Ethiopia: Tikur Anbessa Specialized Hospital (TASH), St. Paul’s Millennium Medical College (SPMMC) and Ghandi Memorial Hospital, located in Addis Ababa, and Jimma University Medical Centre (JUMC) and Gondar University Hospital (GUH), located in the southwestern and northwestern parts of the country, respectively.

### Ethical Approval

Addis Ababa University and all participating institutions provided ethical approval of the study. All parents were provided written informed consent prior to their infants’ participation in the study.

## Laboratory Analysis

### Sample Collection, Isolation and Identification of Bacteria

At all hospitals, a minimum of 1ml of blood was drawn aseptically from the neonates with suspected sepsis using a butterfly needle. At Jimma and St. Paul Hospitals, the blood was added into BD BACTEC Peds Plus blood culture bottle and gently mixed. The blood culture bottle was then incubated in the BD BACTEC^TM^ FX40 machine which was inspected daily for a total of 5 days. When an alert was observed, the BACTEC bottle was taken from the machine, and sub-cultured on MacConkey Blood agar (Oxoid, England), and Chocolate Agar (candle jar) media and incubated at 35-37^°^C for 18-24 hours aerobically. At TASH and GUH, the blood specimens were collected and inoculated on brain heart infusion broth and inspected for five days. At all hospitals, further bacterial identification was performed based on the American Society for Microbiology (ASM) identification guidelines.

### Antimicrobial Susceptibility Testing

Antibiotic susceptibility testing was carried out using the Kirby Bauer disc diffusion technique using Muller Hinton agar (Oxoid, England). Antibiotic discs were selected based on CLSI guidelines. Accordingly, penicillin, cefoxitin, ampicillin, erythromycin, clindamycin, trimethoprim-sulphamethoxazole, oxacillin, amoxicillin clavulanic acid, ceftazidime, gentamicin, ciprofloxacin, and chloramphenicol were used. All the antimicrobials used for the study were obtained from Oxoid Ltd., Basingstoke, Hampshire, UK. The drug susceptibility results were measured using calipers and based on zone of inhibition; the results were reported as resistant, intermediate and susceptible to the treating clinicians so that they could modify the treatment accordingly.

Multi drug resistance (MDR) was defined as bacterial strains resistant to at least one agent in three or more antimicrobial classes.

### Quality Control

Data were checked and reviewed regularly for any incompleteness or outliers and any issues were rectified by the site principal investigators before entry into the database. Reference strains of *Escherichia coli* ATCC 25922 and *S. aureus* ATCC 25923 were used for media and antibiotic disk performance checks.

### Data Analysis and Interpretation

Data were collected using Case Report Forms (CRF) that were formulated and reviewed by various physicians in their respective field of expertise. The data were entered into a computer using the data management system (DMS) developed for this study. Finally, electronic data were transferred, cleaned, merged to one master data and stored centrally using the Structured Query Language database that was later analyzed in SPSS version 20. Frequencies and cross tabulations were used to summarize descriptive statistics. Descriptive statistics were also used to present antimicrobial susceptibility patterns.

## Results

### Clinical Characteristics of the Participants

**Figure 1.**
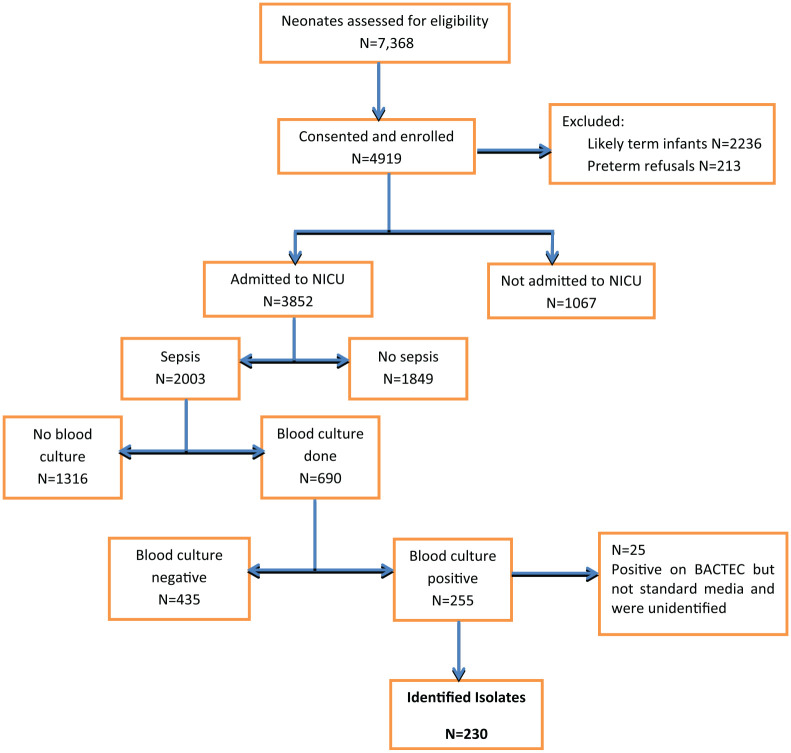
Consort flow diagram.

Among all preterm newborns (n = 4,919) enrolled into the study, 2,003 had a clinical diagnosis of sepsis, making the overall proportion of clinical sepsis 40.7%. Of those diagnosed with clinical sepsis, 1,807 (90.2%) had early onset neonatal sepsis (EONS) and 196 (9.7%) had late onset neonatal sepsis (LONS) (Figure 1).

The majority of those with clinical sepsis were male (53%) and 905 (45%) were female. There were more early (<34 weeks) preterm newborns with a diagnosis of clinical sepsis, 1,071 (53.4%) than late preterm newborns (34-36 weeks), 932 (46.5%). Most were delivered in health institutions (1893,94.5%) either in the study hospitals or health centers.

Of the study participants with neonatal sepsis, only one-third, 690 (34%) had blood cultures done, of which 255 (36.9%) showed bacterial growth (Table 1). Nineteen different bacterial pathogens were identified. Gram-negative bacterial species were the most commonly isolated organisms 134 (58.2%) compared to Gram-positive organisms 90 (39.1%). The most commonly isolated bacteria were *Klebsiella species (spp.)* 78 (33.9%) followed by *Coagulase negative Staphylococcus (CoNS)* 42 (18.2%), *S. aureus* 39 (16.9%) and *E. coli* 12 (5.2%).

**Table 1.** Bacteria Identified in Preterm Newborns Admitted with a Diagnosis of Sepsis to Selected Hospitals in Ethiopia.

Etiologies	N(%)
*Klebsiella spp.*	78(33.9)
S.aureus*	39(16.9)
CoNS*	42(18.2)
E.coli*	12(5.2)
*Serratia spp*	10(4.3)
*Enterobacter spp*	9(3.9)
*Acinetobacter spp*	6(2.6)
*Lactose fermenting rods*	6(2.6)
*Gram-negative rods on BA but not on media*	7(3.0)
*Group B streptococcus*	2(0.8)
Others	19(8.2%)
**Total**	**230**

Others- Pseudomonas aeruginosa, Enterococcus, Streptococcus viridians, Salmonella species, Citrobacter, Bacillus species, mixed colony, Candida spp. *CoNS, Coagulase negative staphylococcus; E. coli, Escherichia *coli; S. aureus, Staphylococcus aureus.*

Gram-positive bacteria showed high resistance to penicillin (98.9%), amoxicillin-clavulanic acid (79.3%) and ceftriaxone (91.3%). *S. aureus* was found to be highly resistant to Oxacillin(85.3%). Better sensitivity to ciprofloxacin was observed among the Gram-positive bacteria (59%) (Table 3). Gram-negative bacteria were highly resistant to the first- and second-line empiric antibiotics such as gentamicin (83.2%) and 3rd generation cephalosporins such as ceftazidime (88%) and ceftriaxone (83.2%). The best sensitivity was observed to ciprofloxacin (65%) (Table 2).

**Table 2.** Antibiotic Resistance Patterns of Isolated Gram-Negative Bacteriapathogens.

Bacterial isolates	Number of isolates	Resistance patterns of isolates to antimicrobial agents								
		CN N(%)	AMP	CAZ	AUG	CHL	TE	CRO	SXT	CIP
*Klebsiella spp.*	75	65(86.6)	75(100)	69(92)	72(96)	63(84)	54(72)	68(90.6)	70(93.3)	34(45.3)
*E. coli*	12	8(66.6)	12(100)	10(83.3)	4(33.3)	7(58.3)	3(23)	10(83.3)	6(50)	2(16.6)
*Serratia spp.*	10	9(75)	10(100)	10(100)	10(100)	3(30)	6(60)	9(90)	9(90)	2(20)
*Enterobacter spp.*	9	8(88.8)	9(100)	8(88.8)	5(55.5)	5(55.5)	3(33.3)	4(44.4)	8(88.8)	3(33.3)
*Acinetobacter spp.*	6	5(83.3)	6(100)	5(83.3)	5(83.3)	5(83.3)	4(66.6)	5(83.3)	5(83.3)	0
Salmonella *spp.*	1	1(100)	1(100)	1(100)	1(100)	0	1(100)	1(100)	0	0
*P. aeruginosa*	1	1(100)	1(100)	1(100)	1(100)	0	1(100)	1(100)	1(100)	0
*Citrobacter spp.*	1	1(100	1(100)	1	1(100)	1(100)	1(100)	1(100)	1(100)	0
*A. xylosoxidans*	1	1(100)	1(100)	1(100)	1(100)	1(100)	1(100)	1(100)	1(100)	0
Unknown bacteria	9	5(55.5)	7(77.7)	4(44.4)	5(55.5)	5(55.5)	6(66.6)	4(44.4)	5(55.5)	3(33.3)
Total	125	83.2		88		72		83.2		35.2

Note: spp. species, CN, gentamicin; AMP, ampicillin; CAZ, ceftazidime; AUG, amoxicillin clavulanic acid; CHL, chloramphenicol; TE, tetracycline; CRO, ceftriaxone; CIP, ciprofloxacin; SXT, sulfamethoxazole-trimethoprim.

**Table 3.** Antibiotic Resistance Patterns of Isolated Gram-Positive Bacterial Pathogens.

Bacteria isolates	Number of isolates	Resistance patterns of isolates to antimicrobial agents									
		P	FOX	ERY	CLN	CN	SXT	TE	CRO	OX	CIP/NOR
*S. aureus*	41	41(100)	35(85.3)	34(82.9)	34(82.9)	25(60.9)	32(78)	33(80.4)	38(92.6)	35(85.3)	24(58.5)
*CoNS*	42	42(100)	39(92.8)	37(88)	37(88)	36(85.7)	37(88)	39(92.8)	40(95.2)	37(88)	29(69)
*GBS*	2	2(100)	2(100)	1(50)	1(50)	0	0	1(50)	2(100)	2(100)	0
*Enterococcusspp.*	3	3(100)	3(100)	3(100)	3(100)	2(66.6)	2(66.6)	3(100)	3(100)	3(100)	2(66.6)
*S. viridians*	2	2(100)	2	1(50)	1(50)	1(50)	1(50)	1(50)	1(50)	2(100)	0
*L. monocytogenes*	1	NA	NA	NA	NA	NA	NA	NA	NA	NA	NA
*Bacillus spp.*	1	NA	NA	NA	NA	NA	NA	NA	NA	NA	NA
Unknown bacteria	3	2(66.6)	1(33.3)	2(66.6)	2(66.6)		1(33.3)	1(33.3)	1(33.3)	1(33.3)	0
Total	95	98.9	88.1	83.8			79.3		91.3	86	59.1

Note: CoNS, Coagulase-negative staphylococci; GBS, group B streptococcus; P, penicillin; FOX, cefoxitin; ERY, erythromycin; CLN, clindamycin; SXT, sulfamethoxazole-trimethoprim; TE, tetracycline; CRO, ceftriaxone; CIP, ciprofloxacin; CN, gentamicin; OX, oxacillin; NA not applicable.

### Multidrug Resistance

Multidrug Resistance (MDR) was observed in 199 isolates (91.3%). *Klebsiella spp.*(92%) and *S. aureus* (92.3%) were found to be predominantly MDR (Table 4).

**Table 4.** Frequency Distribution of Non-MDR and MDR Patterns of Isolated Bacteria.

Bacteria isolates	Number of isolates	MDR N (%)	NMDR N (%)
*Klebsiella spp.*	75	69 (92.0)	6 (8.0)
*S. aureus*	41	38 (92.7)	3 (7.3)
*CoNS*	42	42 (100)	0
*E. coli*	12	9(75.0)	3 (25.0)
*Serratia spp.*	10	10(100)	0
*Enterobacter spp.*	9	8(88.8)	1(11.1)
*Acinetobacter spp.*	6	6(100)	0
*Unknown Gram- negative bacteria*	9	7(77.7)	2(22.2)
*Group B streptococcus*	2	1(50)	1(50)
*Enterococcus spp.*	3	3(100)	0
*S. viridians*	2	1(50)	1(50)
*Unknown Gram-positive bacteria*	3	1(33.3)	2(66.6)
*Salmonella spp.*	1	1(100)	0
*P. aeruginosa*	1	1(100)	0
*Citrobacter spp.*	1	1(100)	0
*A. xylosoxidans*	1	1(100)	0
**Total**	**218**	**199 (91.3)**	**19 (8.7)**

## Discussion

The overall culture positivity rate from patients with symptoms of neonatal septicemia was 36.9%, which was slightly lower than results previously reported from Gondar (46.6%) and Addis Ababa (44.7%).^19^ Higher rates of positive results were reported in Indonesia (65.3%), Georgia (63%) and Yemen (57%).^20-22^

On the other hand, the present study culture positivity rate was higher than observed in studies from Nepal (20.3%) and Gondar (32.1%).^10,23^ In contrast, much lower rates of positive results were reported from Iran (5.6%), Kuwait (8.7%), and Saudi Arabia (5%).^24-26^This variation could be due to methodological differences, time of blood culture collection and prior antibiotic administration, all of which might affect the culture positivity rate.

In our study, the predominant isolates causing sepsis were Gram-negative organisms with *Klebsiella spp.* accounting for 78 (33.9%) followed by Gram-positives like CoNS 42 (18.2%) and *S. aureus* 39 (16.9%). There was a similar report from Addis Ababa where *Klebsiella spp.* (39.2%) and *S. aureus* (22.2%) were the most commonly isolated organisms. A report from Sudan showed that *Klebsiella* (71.1%), *S. aureus* (15.8%), E. coli (5.3%), Gram-positive cocci (2.6%) and *Serratia marcescens* (5.3%) were associated with sepsis in newborns.^27^ In Nigeria as well, neonatal infections were due to Gram-positive and Gram-negative organisms such as *S. aureus* (24.6%), *CoNS* (24.6%) and *Klebsiella spp.* (16.4%).^28^

The results of the current study agree with the reports from most developing countries but differ from those reports from Kuwait where the most frequent organisms isolated were Gram-positive organisms like *Staphylococcus epidermidis* (34%), *Streptococcus viridians* (28%) and *Candida species* (14%). A report from the United Kingdom showed CoNS and *GBS* being organisms frequently seen in EONS, whereas CoNS and *E. coli* were most frequently associated with LONS. A study from the USA also showed that Gram-positive bacteria such as *GBS* were more predominant etiologies of sepsis.^25^

As shown in this report and reports from other developing countries, most pathogens isolated in the hospital setting before 72 hours of life are similar to those isolated afterward. It is highly likely that unclean delivery practices are associated with infections with nosocomial agents very early in life^29^ given that the deliveries of our patients occurred in health facilities (95%).

*Klebsiella spp.* was the predominant isolate in the present study, representing 33.9% of the total isolates, of which *K. pneumoniae* was the predominant bacteria identified. Similar findings reported from Georgia, USA showed *K. pneumoniae* accounting for 29% and Yemen 36.7%.^22^

A very high prevalence of bacterial causes of neonatal sepsis and an alarmingly high level of antibiotic resistance in the isolates were observed in the current study. The overall MDR prevalence was 91.3% and it has been demonstrated that bacteria like *Klebsiella spp.* (92%), *S. aureus (92.7%)* and *CoNS* (100%) were found to be mostly MDR. This frequency is higher than that found from a study done in Gondar where the overall MDR prevalence was 65% and *S. aureus* (61.2%) and *K. pneumoniae* (74%) were found to be the principal MDR strains.^10^ In Addis Ababa, Gram-negative bacteria showed high-level resistance to ampicillin, ceftriaxone, cephalothin, chloramphenicol, and gentamicin. Multidrug resistance was observed in 45.7% and 84.2% of Gram-positive and Gram-negative bacteria respectively.^19^

One of the major limitations of this study is that only about one-third of infants with a clinical diagnosis of sepsis had a blood culture done. Reasons for this gap included failure to order blood culture by the hospital staff as a routine investigation for sepsis and difficulty of obtaining blood encountered in some of the newborns. The other limitation is the different methods of culture used at the different study sites which affects the blood culture positivity, as conventional blood culture suffers from poor sensitivity due to the lack of use of nutritionally enriching substances, inability to neutralize the inherent antimicrobial components in blood and/or in patients receiving antimicrobial treatment.

Although advances in neonatal care have improved survival and reduced complications in preterm infants, sepsis still contributes significantly to mortality and morbidity among very-low-birth-weight infants in neonatal intensive care units. Bacterial isolates from neonatal sepsis show variation from place to place, and indicates that there is a need for continuous monitoring of causative organisms and their drug susceptibility patterns at a local level.

## Conclusion and Recommendations

A higher proportion of bacterial isolates was observed in this study. *Klebsiella spp*, CoNS and *S. aureus* were the predominant pathogens associated with sepsis. The overall prevalence of MDR was 91.3% which was high for most of the isolates. Ciprofloxacin was the most effective drug against both Gram-positive and Gram-negative bacteria. Even though there is a high rate of resistance seen in this report for the commonly prescribed antibiotics, further research is needed at the community level for revision of treatment guidelines.
